# Effect of sodium-glucose cotransporter-2 inhibitors on aldosterone and renin levels in diabetes mellitus type 2 patients: a systematic review and meta-analysis

**DOI:** 10.1038/s41598-022-24280-9

**Published:** 2022-11-15

**Authors:** Worapaka Manosroi, Pojsakorn Danpanichkul, Pichitchai Atthakomol

**Affiliations:** 1 Division of Endocrinology, Department of Internal Medicine, Faculty of Medicine, Chiang Mai University, 110 Intrawarorot Road Soi 2, Si Phum, Amphoe Mueang, Chiang Mai, 50200 Thailand; 2 Faculty of Medicine, Clinical Epidemiology and Clinical Statistics Center, Chiang Mai University, Chiang Mai, Thailand; 3 Department of Microbiology, Faculty of Medicine, Chiang Mai University, Chiang Mai, Thailand; 4 Orthopedics Department, Faculty of Medicine, Chiang Mai University, Chiang Mai, Thailand

**Keywords:** Adrenal gland diseases, Diabetes

## Abstract

The effect of sodium-glucose cotransporter-2 inhibitors (SGLT2i) on plasma aldosterone concentration (PAC) and plasma renin activity (PRA) levels are still inconclusive. This meta-analysis aimed to demonstrate the changes in PAC and PRA levels after the use of SGLT2i in type 2 diabetes patients. A search for relevant publications was performed using *PubMed/Medline*, *Scopus, Cochrane,* and *Embase* databases from their inception through May 2022. Inclusion criteria were studies that contained data on crude PAC and PRA levels before and after the use of SGLT2i in adult type 2 diabetes patients. Standardized mean difference (SMD) with a 95% confidence interval (95% CI) was calculated. Data was separately analyzed by study design: randomized controlled study (RCT) and non-randomized controlled study (non-RCT). Ten studies involving 380 patients were included with two RCT and eight non-RCT. Serum PAC levels showed no significant change after the use of SGLT2i in both RCT and non-RCT. Significantly higher PRA levels were observed after the use of SGLT2i in both RCT and non-RCT with SMD of 0.40 ng/mL/hr; 95% CI (0.06, 0.74) and SMD of 0.36 ng/mL/hr; 95%CI (0.17, 0.55), respectively. Subgroup analysis found significantly higher PRA levels after the use of SGLT2i (SMD 0.45 ng/mL/hr; 95% CI (0.18, 0.71)) only in subgroups that used for three months or less. The use of SGLT2i in diabetes mellitus type 2 patients can affect PRA levels, especially during short-term use. PRA levels should be interpreted with caution in this population.

## Introduction

Sodium-glucose cotransporter-2 inhibitors (SGLT2i), a relatively novel class of oral anti-diabetic agents, have been approved and used in multiple countries as glucose-lowering agents in type 2 diabetes patients as an adjunct to diet and exercise. SGLT2i can be used as a first-line agent, particularly in patients at high risk for atherosclerotic cardiovascular disease (ASCVD) or patients with established major ASCVD^1,2^. In addition to blood glucose control, the use of SGLT2i to reduce mortality in patients with heart failure regardless of whether they have diabetes has also been approved^3^. Other benefits of SGLT2i include weight loss, blood pressure reduction, and reduced hepatic enzymes and hepatic fat in diabetes patients with nonalcoholic steatohepatitis^4,5^. The most commonly reported adverse side effects with SGLT-2-i are genital mycotic infections, urinary tract infections, and increased urination^6^.

SGLT2i can reduce blood glucose by inhibiting sodium-glucose transporter 2 protein which is responsible for glucose coupled with sodium reabsorption in the proximal tubule. It thus prevents the reabsorption of glucose from glomerular filtration and promotes the excretion of glucose in the urine, resulting in lowering blood glucose levels. Apart from a glucosuric effect, SGLT2i can induce natriuresis and osmotic diuresis and can lead to reduced plasma volume and lower blood pressure. These effects can potentially lead to the renin–angiotensin–aldosterone system (RAAS) activation^7^. However, available data regarding the relationship between SGLT2i and RAAS activation are still inconclusive and contradictory. One study suggested that SGLT2i does not affect plasma aldosterone concentration (PAC), plasma renin activity (PRA), or aldosterone-to-renin ratio (ARR)^8^. Another study reported that there is an elevation of PRA but not PAC after the use of SGLT2i^9^. ARR is commonly used to screen for primary aldosteronism (PA). According to the current guidelines for PA diagnosis, to reduce the interpretation interference of ARR, it is recommended that some anti-hypertensive medications, especially mineralocorticoid receptor blockers and potassium wasting diuretics, should be discontinued at least four weeks before the measurement of ARR. Additionally, some anti-hypertensive medications, e.g., angiotensin-converting enzyme inhibitors (ACEI), angiotensin receptor blockers (ARB), dihydropyridine calcium channel blockers, and ß-blockers should be discontinued at least two weeks before the measurement. These medications should be replaced with anti-hypertensive medications with less effect on ARR such as α-blockers, verapamil slow release, and hydralazine^10^. Whether SGLT2i should be discontinued before ARR testing or not is still uncertain.

Patients with PA usually have a higher risk of having glycemic abnormalities than essential hypertension patients, including diabetes mellitus and impaired glucose tolerance^10^. For that reason, the use of SGLT2i in patients suspected of having PA may be common. Determining the best method to maximize the accuracy of laboratory assessments of PAC, PRA, and ARR following alterations resulting from starting SGLT2i in diabetes mellitus type 2 patients is an issue that needs to be addressed. This systematic review and meta-analysis aimed to examine the effect of SGLT2i on PAC and PRA in patients with diabetes mellitus type 2.

## Materials and methods

### Search strategy and selection criteria

This study followed the Preferred Reporting Items for Systematic Reviews and Meta-analyses (PRISMA) guidelines^11^. The predefined protocol was registered in INPLASY 202260050. A comprehensive search of four databases, *PubMed/Medline*, *Scopus*, *Cochrane,* and *Embase*, was performed from their inception through May 2022. The keywords used were “sodium-glucose transporter 2 inhibitors OR SGLT2i” AND “aldosterone OR renin OR hypertension OR plasma volume OR volume OR diabetes”. Medical subject heading (MeSH) terms and Emtree (Embase subject headings) were used in the *PubMed*/*Medline* and *Embase* search. Details of the keywords for each database search are provided in the Supplementary Appendix. References from the included studies, other relevant publications, and non-included reviews were identified and included as additional studies for the initial screening. The duplicate removal and initial screening of abstracts and titles were performed by Rayyan, a web-based program (Rayyan Systems Inc., Cambridge, MA, USA)^12^.

Two authors (WM, PA) independently performed the searches and screened for titles and abstracts. Relevant studies were retrieved and underwent full-text screening based on the inclusion criteria. Then two authors (WM, PD) independently conducted the data extraction and two authors (WM, PA) assessed the methodological quality of the included studies. In cases of disagreement during the article search and selection processes, all three authors discussed the issue and reached a final consensus.

Inclusion criteria for included articles were as follows: (1) prospective or retrospective non-randomized controlled study (non-RCT) or randomized controlled trial study (RCT) performed in adult diabetes mellitus type 2 patients, (2) the study should provide the crude data of plasma renin, either PRA or plasma renin concentrations which can be converted to PRA and/or plasma PAC and/or ARR before and after the use of SGLT2i. The exclusion criteria were (1) studies that stated that anti-hypertensive medications which interfered with PAC or PRA measurement were allowed to be adjusted during the study period, (2) articles published in a language other than English, review articles, case reports, grey literature, editorial comments, conference abstracts and animal studies and (3) studies involving special populations such as pregnant women or children.

### Data extraction

Data extraction was independently conducted by two authors (WM and PD) using predesigned Microsoft Excel spreadsheets. The demographic variables extracted from the studies were: (1) study characteristics, i.e., the name of the first author, year of publication, the country where the study was conducted, study design, number of patients, whether the doses of anti-hypertensive medications were stabilized during the study period, (2) patient characteristics and potential confounders, i.e., means and standard deviations (SD) of age, percentage of males, percentage of patients with hypertension, means and SD of glycated hemoglobin (HbA1c), estimated glomerular filtration rate (eGFR), means and SD of body mass index (BMI), and systolic and diastolic blood pressure, (3) SGLT2i data including type and dose of SGLT2i and duration of use and (4) outcomes of interest including mean PAC, PRA and ARR levels, both before and after the administered of SGLT2i. In RCT studies, the demographic data and data on PAC and PRA levels were retrieved only from the SGLT2i arm. If plasma renin concentrations were presented in the study, they were converted to PRA level with the unit of ng/mL/hr^10^. For PAC data, the units were converted to ng/dL.

### Data synthesis

The meta-analysis was performed using the STATA program version 16.0. The data was analyzed separately by study type (non-RCT and RCT). Standardized mean difference (SMD) with a 95% confidence interval (CI) was calculated for the changes in levels of PAC and PRA during the period of use of SGLT2i. Random-effect modelling was performed in this study as the observed estimates of treatment effects can vary across studies due to real differences in the treatment effects in each study and sampling variability. The statistical significance level for this meta-analysis was set at *p *< 0.05. To evaluate the statistical heterogeneity among the studies, the I^2^ statistic was assessed. I^2^ values of > 75% with a significant Cochran Q test (*p *< 0.05) were considered to indicate high heterogeneity. I^2^ values of < 25–50% and > 50–75% were considered as low and moderate heterogeneity, respectively. Publication bias was assessed using funnel plots and Egger’s linear regression test. A p-value of < 0.05 was set to indicate statistically significant publication bias, as well as the presence of asymmetry of a funnel plot.

Subgroup analysis was also conducted to determine the effect of potential confounders which included duration of SGLT2i use, and whether the dose of anti-hypertensive medications was stabilized during the study period. The duration of SGLT2i use was classified as either short duration (≤ 3 months) or long duration (> 3 months) based on a study demonstrating that SGLT2i can alter eGFR within three months after use and will return to a normal level three months after use^13^. For subgroup analysis by anti-hypertensive medication dosage, groups were categorized as either stable doses during the study or as unknown dose status. Studies that mentioned the allowance of dose adjustment of anti-hypertensive medications were primarily excluded. As the aim of this meta-analysis was to focus on the changes in levels of PAC and PRA, studies that concomitantly used SGLT2i and anti-hypertensive medications which can interfere with PAC and PRA measurement were included as long as the studies did not mention the allowance of anti-hypertensive medication dose adjustment during the study.

### Risk of bias assessment

Assessment of the risk of bias was performed independently by two authors (WM and PA) using the ROBINS-I tool (Risk of Bias In Non-randomized Studies-of Interventions) for a non-RCT studies^14^. Version 2 of the Cochrane tool for assessing the risk of bias in randomized trials (RoB 2) was employed to assess the risk of bias in RCT studies^15^. Any disputes were resolved by the third author (PD).

### Certainty of the evidence

The quality of the evidence was graded independently by two authors (WM and PA) using the Grading of Recommendation, Assessment Development and Evaluation (GRADE) methodology^16^. Grading began with an assessment of the quality of the study design which was low for observational studies or non-RCT studies and high for RCT studies. Also, the certainty of the evidence was considered to be increased or decreased based on multiple aspects, e.g., study limitations, consistency of effect, imprecision, indirectness, publication bias, the magnitude of effect, dose–response gradient, and whether any plausible confounders could alter the effect. The certainty of the evidence was classified as “high”, “moderate”, “low” or “very low”. Any conflicts were resolved by the third author (PD).

## Results

**Figure 1:**
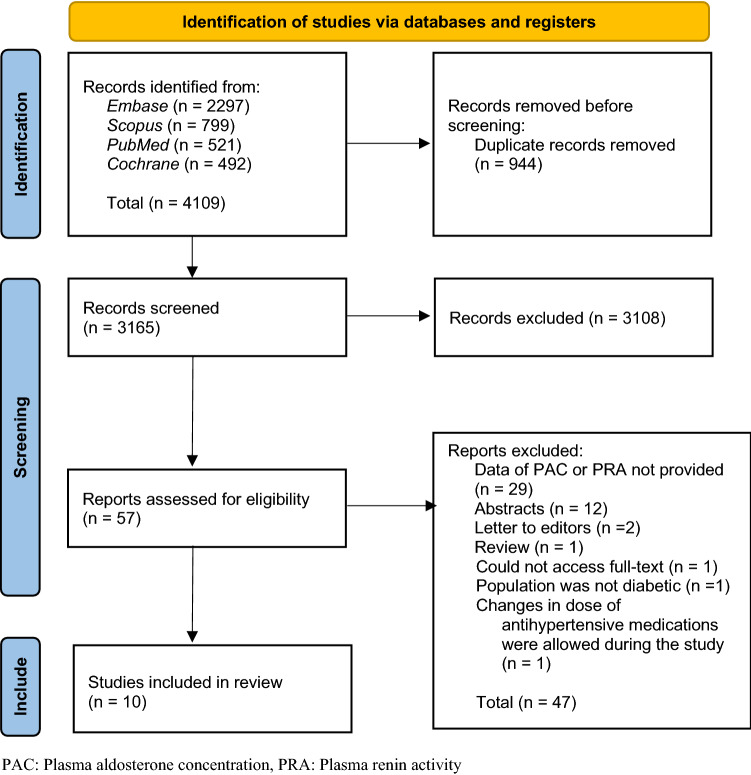
PRISMA Flow Diagram.

A total of 4,109 articles were retrieved from the database searches including 2,297 from *Embase*, 799 from *Scopus*, 521 from *PubMed,* and 492 from *Cochrane*. From the retrieved articles, 944 duplicates were removed. Screening of titles and abstracts of 3,165 articles was performed which resulted in the exclusion of 3,108 which were not relevant to the objectives of this study. The full texts of the remaining 57 articles were retrieved and reviewed, resulting in the exclusion of an additional 47 articles due to multiple reasons including that data on PAC or PRA were not reported, populations were not diabetes mellitus type 2, studies reported allowing change of anti-hypertensive medication dosage during the study. In addition, conference abstracts, letters to the editor, review articles, and reports where the full text was not accessible were also excluded. Ultimately, a total of ten studies were included^9,17–25^. The study selection process is shown in Fig. 1.

### Study characteristics

Table 1 shows the characteristics of the included studies. Among the ten studies included, two were RCT^9,17^, five were prospective studies^19,22–25^, and three were retrospective studies^8,18,21^. The majority of the studies were conducted in Asia, primarily in Japan. Most of the studies were male predominant. Two studies did not provide data on hypertensive prevalence^18,25^. One study did not report data on mean HbA1c and mean BMI^24^. Two studies did not report data on mean systolic and diastolic blood pressure^18,25^, while one study did not report data on mean eGFR^23^. Among the studies, six types of SGLT2i were used: empagliflozin, ipragliflozin, canagliflozin, dapagliflozin, tofogliflozin, and luseogliflozin. One study used multiple types of SGLT2i^8^, while all the others used only one SGLT2i each. The duration of SGLT2i use ranged from two days to 6.5 months. Three studies stated that the dosage of anti-hypertensive medications was stable during the study period^8,9,19^. All ten studies provided data on PAC and PRA levels. One study reported ARR results^8^.

**Table 1:** Characteristics of included studies.

Author (year)	Country	Study design	Number of patients	SGLT2i (mg/day)	Duration of SGLT2i use	Stable dose of antihypertension medications	Age (mean + SD)	Male (%)	HTN (%)	HbA1c% (mean + SD)	eGFR (mL/min/1.73m^2^) (mean + SD)	BMI (mean + SD)	SBP (mean + SD)	DBP (mean + SD)	Overall risk of bias
Higashikawa^18^	Japan	Retrospective	56	Tofoglifozin (20)	3 months	N/A	82.0 ± 6.5	24	N/A	7.4 ± 1.3	60.5 ± 23.2	23.2	N/A	N/A	Low*
Ghanim^19^	USA	Prospective	24	Dapaglifozin (10)	3 months	Yes	61 ± 2	58	75	6.9 ± 0.1	89 ± 5.0	39.1 ± 1.8	134 ± 3	78 ± 2	Moderate*
Sawamura^8^	Japan	Retrospective	40	Empagliflozin (10), Ipragliflozin (50), Canagliflozin (100), Dapagliflozin (5), Tofogliflozin (20), Luseogliflozin (2.5)	2–6 months	Yes	58.3 ± 12.7	50	100	8.1 ± 1.3	81.3 ± 21.3	29.4 ± 5.5	130 ± 13.0	80 ± 10	Moderate*
Kataoka^21^	Japan	Retrospective	10	Empagliflozin (10)	1–2 months	N/A	61.7 ± 14	40	70	8.55 ± 0.9	76.3 ± 14.4	32.1 ± 3.7	128 ± 9.83	75.8 ± 9.0	Low*
Isshiki^9^	Japan	Randomized controlled trial	47	Dapagliflozin (5)	6 months	Yes	64.7 ± 6.3	53.2	47	7.33 ± 0.5	87.7 ± 22.3	26.4 ± 3.7	138.7 ± 16.7	75.2 ± 11.7	Some concerns^#^
Solini^17^	Italy	Randomized controlled trial	20	Dapagliflozin (10)	1 month	N/A	60 ± 8	60	100	7.5 ± 3.5	94.7 ± 11.9	32.38 ± 6.7	135.9 ± 11.4	75.6 ± 6.5	Some concerns^#^
Griffin^22^	Ireland	Prospective	20	Empagliflozin (10,25)	6.5 months	N/A	58.45 ± 8.9	80	70	8.6 ± 3.4	94 ± 13.0	31 ± 5.5	133 ± 13.0	75 ± 9	Low*
Nomiyama^23^	Japan	Prospective	134	Ipragliflozin (50)	6 months	N/A	53.9 ± 10.5	52.2	50.8	8.02 ± 1.2	N/A	29.6 ± 4.9	133.5 ± 15.9	79.3 ± 11.8	Low*
Solini^24^	Italy	Prospective	16	Dapagliflozin (10)	2 days	N/A	57 ± 9	68.7	0	N/A	95.7 ± 12.8	N/A	130.6 ± 12.8	75.3 ± 6.3	Low*
Tanaka^25^	Japan	Prospective	13	Canagliflozin (100)	6 days	N/A	51.2 ± 9.4	76.9	N/A	8.06 ± 0.7	81.9 ± 20.6	26.7 ± 4.3	N/A	N/A	Low*

*N/A* Data not available, *HTN* Hypertension, *BMI* Body mass index, *SBP* Systolic blood pressure, *DBP* Diastolic blood pressure.

*Assessed by ROBINS-I tool. ^#^Assessed by RoB 2.

### Risk of bias in the studies

The risk of bias was assessed using the ROBINS-I tool for non-RCT studies (Table 1). Two studies evidenced a moderate risk of bias^8,19^. The other six studies evidenced a serious risk of bias due to one major reason, an important confounder: the stable dosage of anti-hypertensive medications during the study period was not stated or appropriately controlled for by proper statistical analyses. For RCT studies, RoB 2 was used and all of the studies showed some concerns risk of bias. Details of the risk of bias scores for each study are shown in the Supplementary Appendix.

### Results of syntheses and subgroup analysis

**Figure 2:**
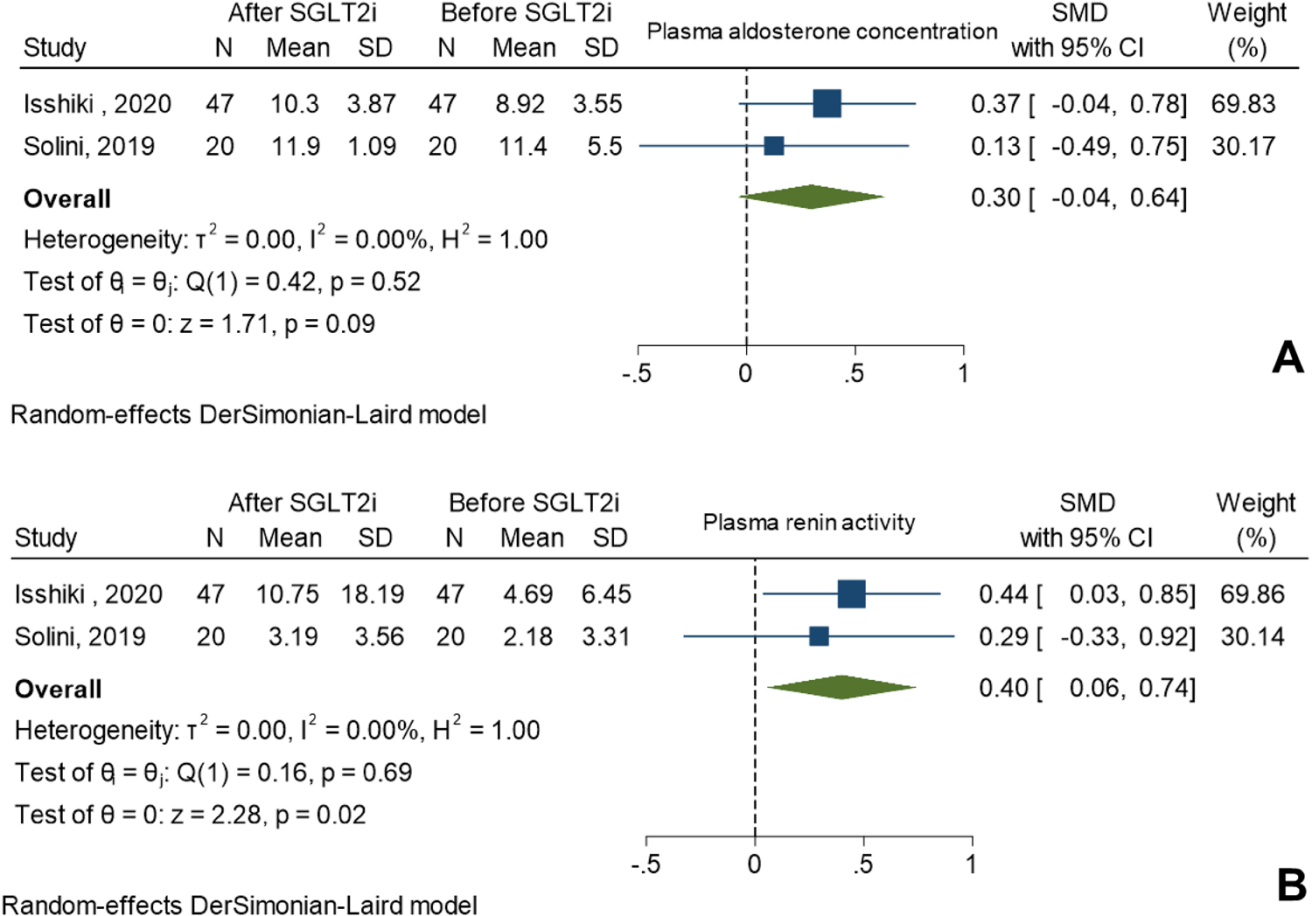
Forest plots of changes in (**A**) plasma aldosterone concentration and (**B**) plasma renin activity after the use of SGLT2i in randomized controlled studies.

**Figure 3:**
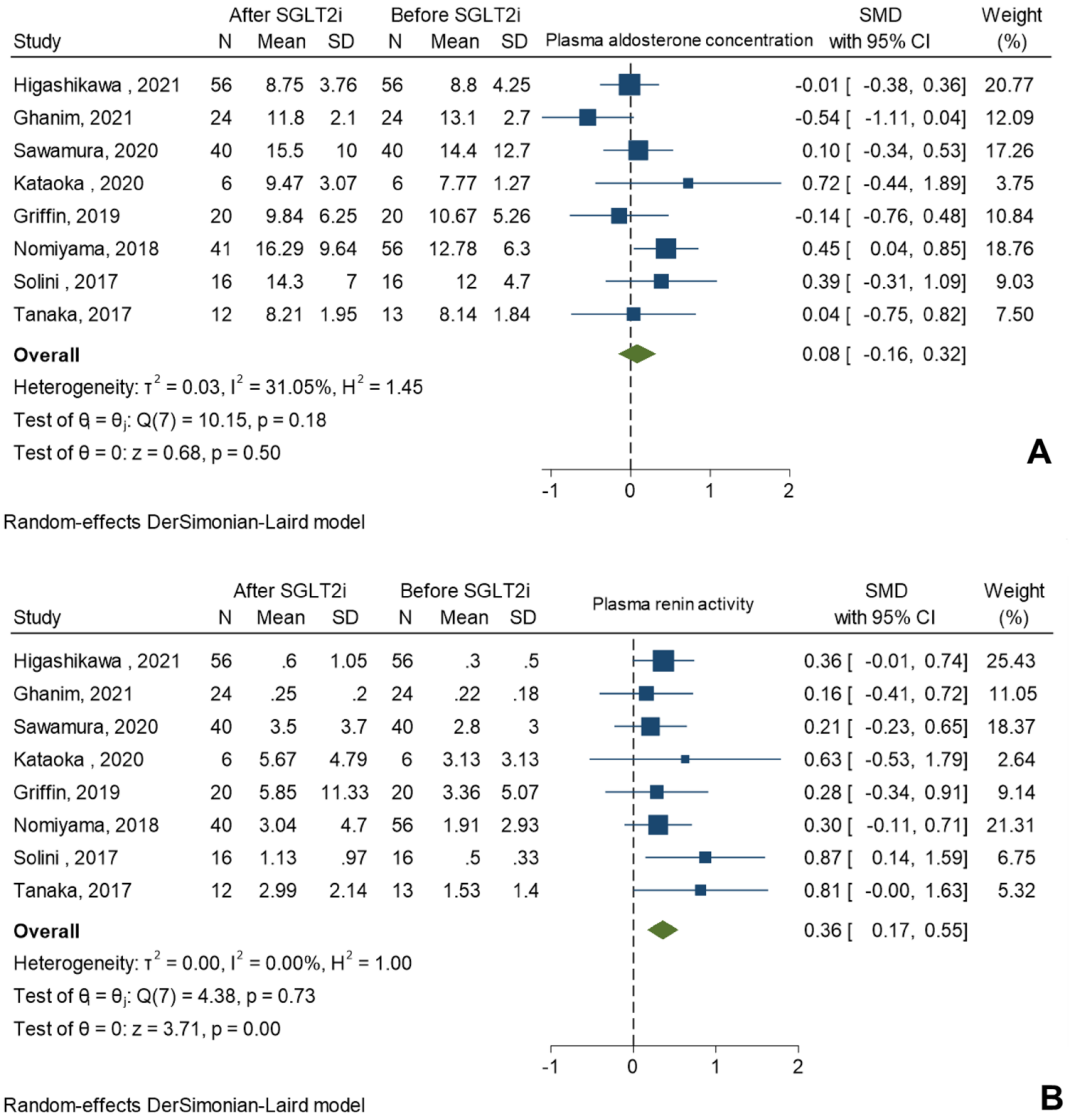
Forest plots of changes in (**A**) plasma aldosterone concentration and (**B**) plasma renin activity after the use of SGLT2i in non-randomized controlled studies.

A total of ten studies comprising 380 patients were included in this meta-analysis. There were 67 patients in two RCT studies while there were 313 patients in eight non-RCT studies. Serum PAC level showed no significant changes after the use of SGLT2i in both RCT and non-RCT studies, with SMD of 0.30 ng/dL; 95% CI (− 0.04, 0.64) and SMD of 0.08 ng/dL; 95%CI (− 0.16, 0.32), respectively. For PRA, a significantly higher level was observed after the use of SGLT2i in both RCT and non-RCT studies with SMD of 0.40 ng/mL/hr ; 95% CI (0.06, 0.74) and SMD of 0.36 ng/mL/hr; 95%CI (0.17, 0.55), respectively. The forest plots are shown in Figs. 2 and 3. The meta-analysis could not be performed for ARR because there was only one study that provided results after using SGLT2i^8^. The crude result for ARR in this study showed that there was no significant changes of ARR after the use of SGLT2i (SMD of − 0.21 ng/dL per ng/(mL·hr); 95%CI (− 0.65, 0.23).

Subgroup analysis was only carried out in eight non-RCT studies because there were only two RCT studies included. In the subgroup analysis of PAC level by duration of SGLT2i use, and whether anti-hypertensive medications dosage was stabilized during the study, there were no significant changes in PAC level after SGLT2i use among the aforementioned subgroups. In the subgroup analysis of PRA level by duration of SGLT2i use, only the subgroup which use ≤ 3 months showed a significantly higher PRA level after the use of SGLT2i (SMD 0.45 ng/mL/hr; 95% CI (0.18, 0.71)). Only studies that did not mention stabilization of anti-hypertensive medications dosage demonstrated higher PRA after the use of SGLT2i (SMD 0.43 ng/mL/hr; 95% (0.20, 0.65). Data are as shown in Table 2 and the Supplementary Appendix.

**Table 2:** Subgroup analysis in non-randomized controlled studies.

Subgroup	No. of analyses	SMD (95% CI)	I^2^ (%)	I^2^ *p*-value
**Plasma aldosterone concentration (ng/dL)**				
Duration				
> 3 months	3	0.19 (− 0.14, 0.51)	28.17	0.25
≤ 3 months	5	0.00 (− 0.35, 0.35)	33.03	0.20
Stable dose of antihypertensive medications				
Unknown status	6	0.18 (− 0.04, 0.40)	0.03	0.42
Yes	2	− 0.19 (− 0.81, 0.43)	66.04	0.09
**Plasma Renin Activity (ng/mL/hr)**				
Duration				
> 3 months	3	0.26, (− 0.01, 0.53)	0	0.95
≤ 3 months	5	0.45 (0.18, 0.71)	0	0.50
Stable dose of antihypertensive medications				
Unknown status	6	0.43 (0.20, 0.65)	0	0.50
Yes	2	0.19 (− 0.16, 0.54)	0	0.73

### Reporting of heterogenicity

Low heterogeneity among the studies was observed for both PAC and PRA in RCT studies with I^2^ values of 0% and 0%, respectively. Also, in non-RCT studies, low heterogeneity among the studies was observed for both PAC and PRA with I^2^ values of 31.05% and 0%, respectively.

### Publication bias

Egger’s regression test did not reveal any publication bias for PAC and PRA in non-RCT studies with p-values of 0.675 and 0.208, respectively. Similarly, the funnel plots for both PAC and PRA were symmetrical (Supplementary Appendix). In RCT studies, publication bias cannot be assessed due to the small number of included studies.

### Certainty of the evidence

In RCT studies, GRADE assessment for the certainty of the evidence found the outcome of PAC showed moderate certainty of the evidence. The studies on PAC outcome were considered imprecise due to wide CI with threshold crossing (downgraded one levels). For that reason, the overall certainty for PAC was moderate. There was no imprecision for PRA outcome among the studies as there was with PAC. The overall certainty for PRA was considered high.

In non-RCT studies, GRADE assessment for the certainty of the evidence found the outcome of PAC showed low certainty of the evidence because the studies were non-RCT. As the ROBINS-I tool was used to assess the risk of bias, most of the studies on PAC outcome had a serious risk of bias and were considered imprecise due to wide CI with threshold crossing (downgraded two levels). For that reason, the overall certainty for PAC was low. There was no imprecision for PRA outcome among the studies as there was with PAC. The overall certainty for PRA was considered moderate (Supplementary Appendix).

## Discussion

This systematic review and meta-analysis demonstrated the changes in PAC and PRA after the use of SGLT2i in diabetes mellitus type 2 patients. There were no significant changes in PAC after the use of SGLT2i, while a significantly increased level of PRA was observed in those patients. These results were observed in both RCT and non-RCT studies. For PRA, a significantly increased level was observed only with short-term use of SGLT2i (≤ 3 months) while there was no significant change with chronic use of SGLT2i (> 3 months). The findings of this meta-analysis can be applied to laboratory interpretation of PAC and PRA during PA investigations, especially in diabetes patients who are currently receiving SGLT2i treatment. The mildly increased PRA levels with short-term SGLT2i use can cause falsely low ARR.

SGLT2i can increase osmotic diuresis and natriuresis, especially during the early phase of treatment. The reduction of renal perfusion pressure and sodium load at the distal tubule can activate systemic RAAS by stimulating renin release from juxtaglomerular cells^26^. Conflicting results of intrarenal RAAS were reported in animal studies. One animal study stated that intrarenal RAAS can be activated to compensate for the loss of sodium and water after the use of SGLT2i^27^, while another study showed decreased intrarenal RAAS activation^28^. Decreased angiotensin (AGT) production in the proximal tubule could be the result of a reduction in blood glucose level due to SGLT2i action. Contrariwise, SGLT2i can increase glucose delivery to the distal tubule which can stimulate AGT production, suggesting that different types of SGL2i may have different effects on glucose lowering and can cause inconsistent results of intrarenal RAAS in response to SGLT2i^29^.

A range of findings regarding changes in PAC and PRA after SGLT2i use were found. One animal study using a diabetic rat model found that the use of SGLT2 for 10–12 weeks caused increased levels of PAC and PRA during the acute phase but that the levels remained unchanged with chronic use of SGLT2i^27,28^. Clinical studies also provided conflicting results. After 12 weeks of SGLT2i treatment, increases in PAC and PRA were found in one study, while another study reported no significant changes in either PAC or PRA^30,31^. These two studies were not included in our meta-analysis, however, as they did not meet the inclusion criteria. The former study did not report the crude data of PAC and PRA and the latter study was an abstract presentation.

The underpinning pathophysiology of the observed results for PAC and PRA can be explained by multiple theories. No observed changes of PAC can be explained by increased aldosterone production which was not stimulated solely from AngII which resulted from volume reduction. Other factors such as adrenocorticotrophic (ACTH) hormone, serum potassium, and the patient’s posture can also increase the production of PAC. In addition, the time when the sample is collected, and circadian variation can influence aldosterone secretion^32^. Significantly increased PRA levels after SGLT2i treatment can be directly explained by transiently systemic RAAS activation. However, this change was observed to be only temporarily during the early phase of treatment based on subgroup analysis results. Various research studies reported the diuretic action and natriuretic effect of SGLT2i were transient and were no longer observed after a few days or a few months, with the duration varying in different studies^25,33,34^.

Subgroup analysis showed that significantly increased levels of PRA after SGLT2i use were found only in studies that did not provide information regarding the adjustment of anti-hypertensive medications, while studies that specified no adjustment of medications during the study reported no significant changes in PRA. An increased dose of anti-hypertensive medications, e.g., diuretics, ACEI, ARB, and dihydropyridine calcium channel blockers, can cause an elevation of PRA. Therefore, the results of this meta-analysis should be interpreted with caution.

This meta-analysis has multiple strengths. First, to the best of our knowledge, this meta-analysis is the first to report the effect of SGLT2i in the alteration of PAC and PRA levels, information important to the diagnosis of and screening for PA. Second, the included studies showed low heterogeneity which means there was low between-study variation. These strengths suggest the results can be employed in the general population in real-world practice. In terms of practical use, if elevated PRA which can cause falsely low ARR is observed in those who use SGLT2i within the first three months, a repeat test of PRA should be performed after SGLT2i discontinuation. However, if PRA is suppressed during the use of SGLT2i, repeat testing is unnecessary. Another strength is that subgroup analysis was performed to identify the effects of possible confounders.

Some limitations in this study need to be acknowledged. First, only one study reported ARR result after the use of SGLT2i^8^, so a meta-analysis of ARR could not be conducted. However, we have performed exploratory analysis of this only study which revealed that there was no significant changes of ARR after the use of SGLT2i. Research on ARR changes after SGLT2i treatment should be conducted in the future. Second, whether dose adjustment of anti-hypertensive medications was allowed during the study period was not mentioned in most of the included studies. Consequently, whether the changes in PRA were the effect of adjustments to anti-hypertensive medications during the study could not be addressed. Third, for most of the included studies, their primary aims did not include an investigation of changes in PAC and PRA levels. Thus, strict protocol during the collection of PAC and PRA might not have been applied, e.g., daily sodium intake or patient’s posture might have interfered with the results. Fourth, this meta-analysis included only diabetes patients who used SGLT2i as there was only one study that reported PAC and PRA results in non-diabetic patients. Further study of SGLT2i use and the effect of RAAS in non-diabetic patients is warranted. Lastly, most of the studies had a serious risk of bias, so interpretation of the results should be done with caution.

## Conclusion

The use of SGLT2i in diabetes mellitus type 2 patients does not affect PAC, but it can increase PRA levels. Clinical practitioners should interpret PAC and PRA results cautiously during the screening for and diagnosis of PA, particularly in diabetes patients undergoing SGLT2i treatment. A future study regarding changes in ARR following the use of SGT2i is warranted.

## Supplementary Information

Supplementary Information.

## Data Availability

The datasets used and/or analyzed during the current study are available upon request.
