# Supplementary material for: Effect of sodium-glucose cotransporter-2 inhibitors on aldosterone and renin levels in diabetes mellitus type 2 patients: a systematic review and meta-analysis
Source: Sci Rep. 2022 Nov 15;12:19603. doi: 10.1038/s41598-022-24280-9 (PMC9666660; doi:10.1038/s41598-022-24280-9)
Supplement: Supplementary file 1 — Supplementary Information. [file 41598_2022_24280_MOESM1_ESM.docx]

**Supplementary appendix**

**Table S1.** Keywords for article searches

**Table S2.** Grading of Recommendations Assessment, Development and Evaluation (GRADE) rating in randomized controlled studies

**Table S3.** Grading of Recommendations Assessment, Development and Evaluation (GRADE) rating in non-randomized controlled studies

**Figure S1.** Overall risk of bias assessed using RoB 2 for randomized controlled studies

**Figure S2.** Overall risk of bias assessed using ROBINS-I tool for non-randomized controlled studies

**Figure S3.** Forest plots of subgroup analysis of changes of plasma aldosterone concentration after SGLT2i use in non-randomized controlled studies by (A) study type (B) duration of SGLT2i use and (C) the constancy of the dosage of anti-hypertensive medications during the study

**Figure S4.** Forest plots of subgroup analysis of the changes of plasma renin activity after SGLT2i use in non-randomized controlled studies by (A) study type (B) duration of SGLT2i use and (C) the constancy of the dosage of anti-hypertensive medications during the study

**Figure S5.** Funnel plots in non-randomized controlled studies (A) plasma aldosterone concentration (B) plasma renin activity

**Table S1** Keywords for article searches

| Keywords | Number of articles |
| --- | --- |
| *Embase* | 2297 |
| 'sodium glucose cotransporter 2 inhibitor'/exp AND ('aldosterone'/exp OR 'renin'/exp OR 'plasma volume'/exp OR 'hypertension'/exp) |  |
| *Scopus* | 799 |
| (("Sodium-Glucose Transporter 2 Inhibitors") AND ("Aldosterone" OR "Renin" OR "Hypertension" OR "Plasma Volume" OR volume)) |  |
| *PubMed* | 519 |
| (("Sodium-Glucose Transporter 2 Inhibitors"[Mesh]) AND ("Aldosterone"[Mesh] OR "Renin"[Mesh] OR "Hypertension"[Mesh] OR "Plasma Volume"[Mesh] OR volume)) |  |
| *Cochrane* | 492 |
| "Sodium-Glucose Transporter 2 Inhibitors") AND ("Aldosterone" OR "Renin" OR "Hypertension" OR "Plasma Volume" OR volume)) |  |

**Table S2.** Grading of Recommendations Assessment, Development and Evaluation (GRADE) rating in randomized controlled studies

| **Outcome** | **Certainty assessment** | | | | | | | **Absolute effect (95% CI)** | **Certainty** |
| --- | --- | --- | --- | --- | --- | --- | --- | --- | --- |
|  | **No. of studies** | **Study design** | **Risk of bias** | **Inconsistency** | **Indirectness** | **Imprecision** | **Other considerations** |  |  |
| Plasma aldosterone concentration | 2 | RCT | Not serious | Not serious | Not serious | Serious | None | 0.30 (-0.04, 0.64). | Moderate |
| Plasma renin activity | 2 | RCT | Not serious | Not serious | Not serious | Not serious | None | 0.40 (0.06, 0.74) | High |

**Table S3.** Grading of Recommendations Assessment, Development and Evaluation (GRADE) rating in non-randomized controlled studies

| **Outcome** | **Certainty assessment** | | | | | | | **Absolute effect (95% CI)** | **Certainty** |
| --- | --- | --- | --- | --- | --- | --- | --- | --- | --- |
|  | **No. of studies** | **Study design** | **Risk of bias** | **Inconsistency** | **Indirectness** | **Imprecision** | **Other considerations** |  |  |
| Plasma aldosterone concentration | 8 | Non-RCT | Serious | Not serious | Not serious | Serious | None | 0.08 (-0.16, 0.32). | Low |
| Plasma renin activity | 8 | Non-RCT | Serious | Not serious | Not serious | Not serious | None | 0.36 (0.17, 0.55) | Moderate |


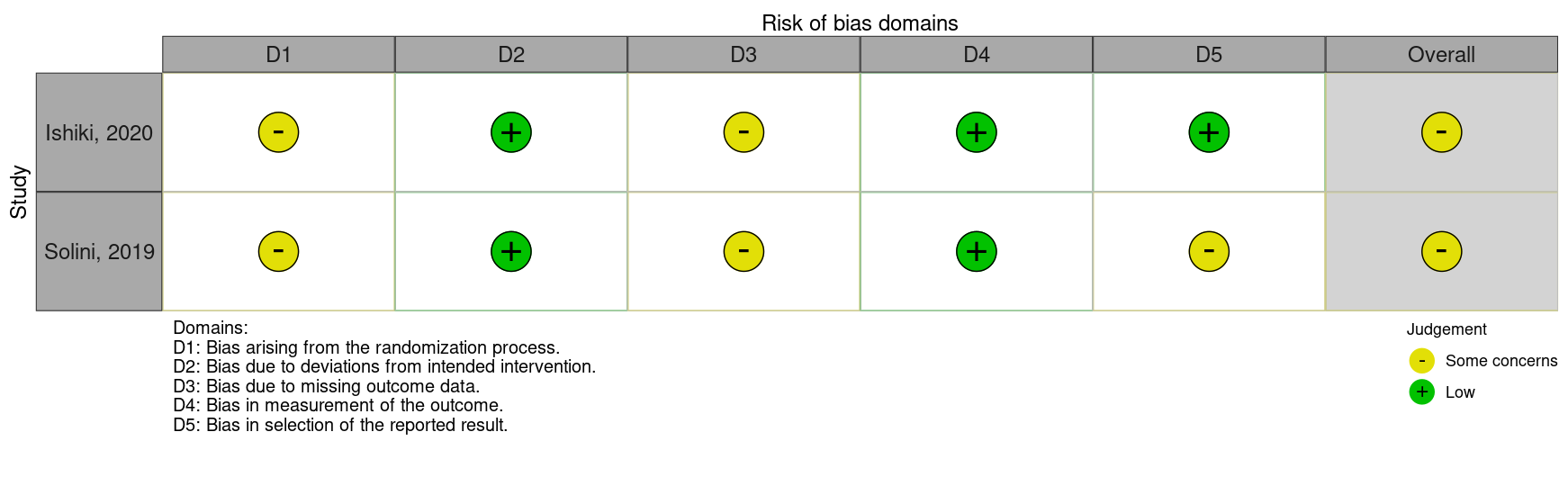


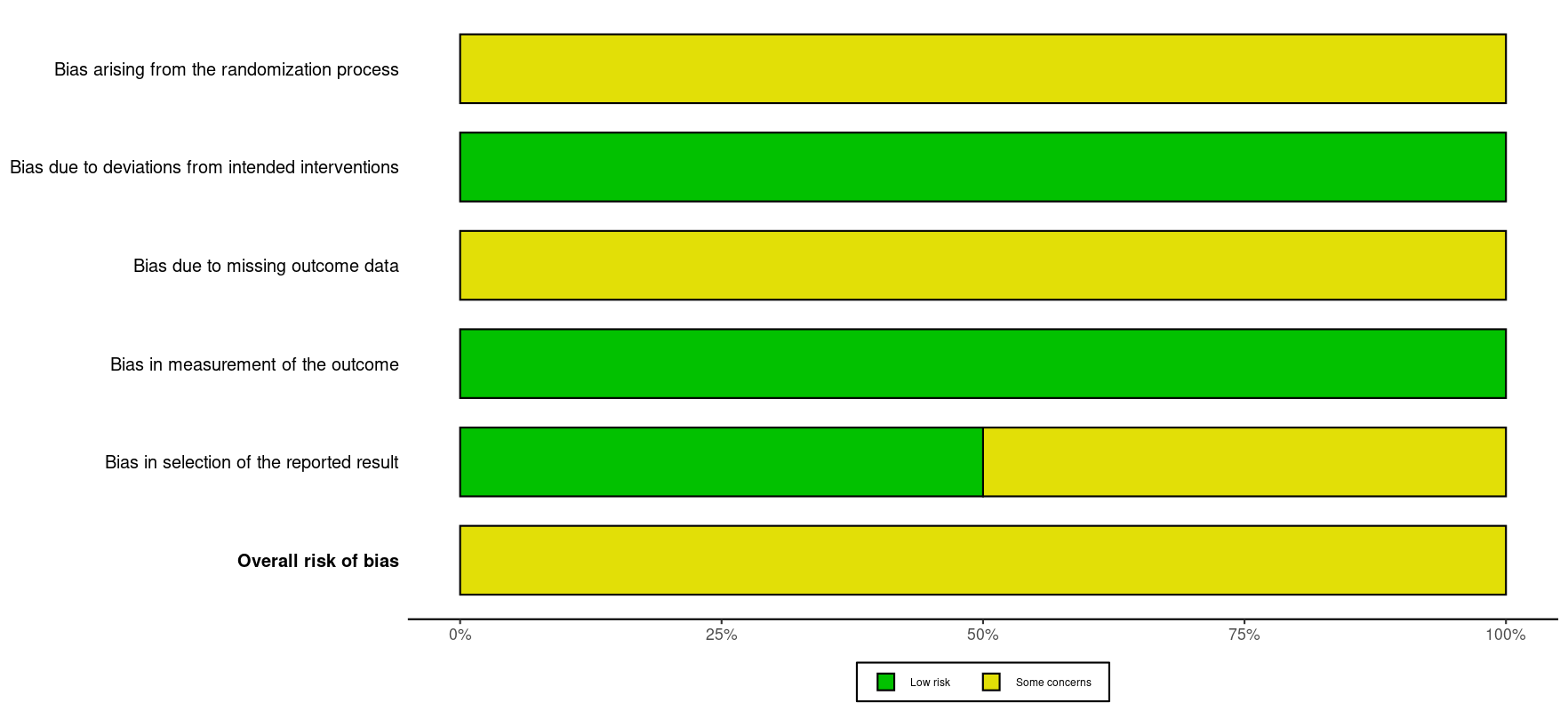


**Figure S1.** Overall risk of bias assessed using RoB 2 (A) traffic light plot and (B) summary plot


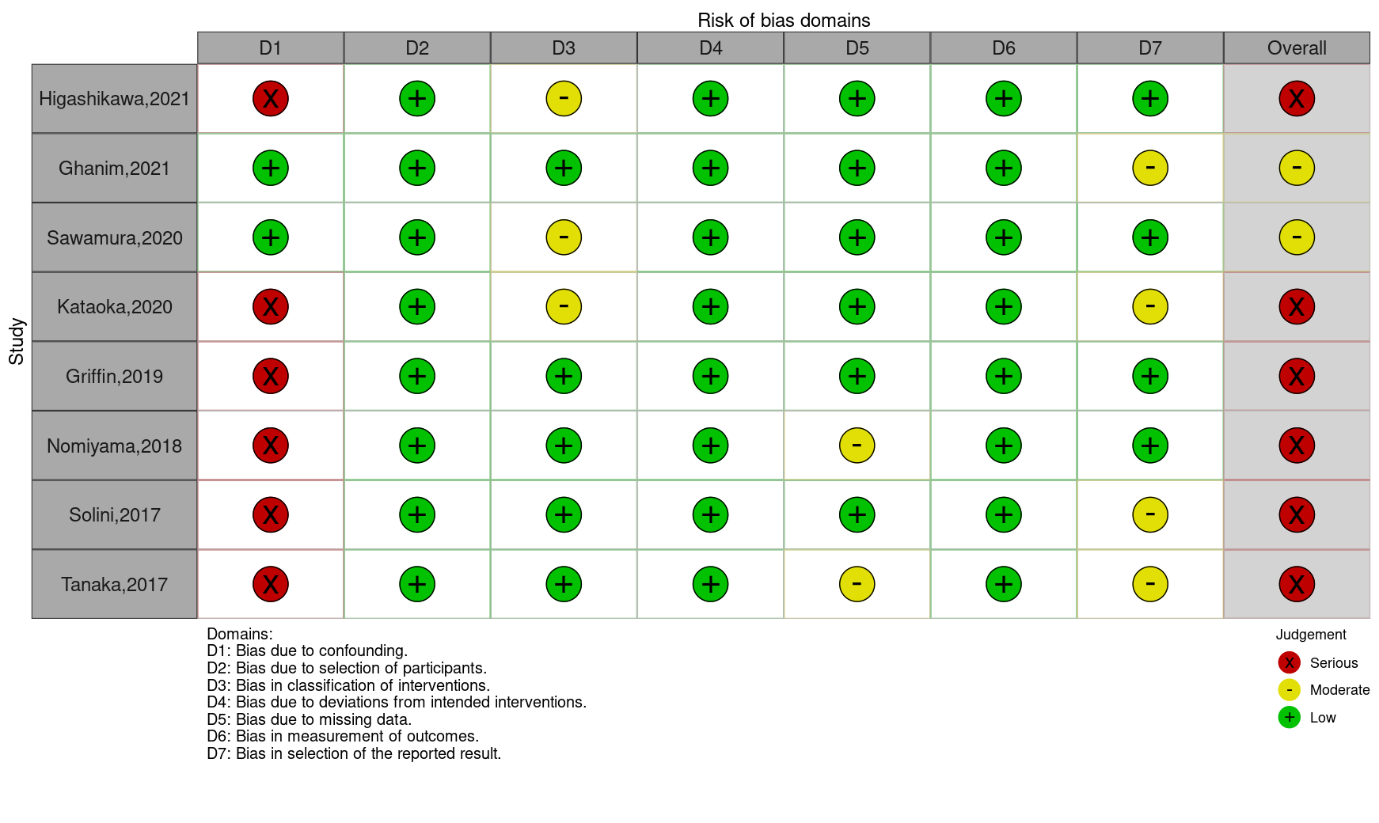


**A**

**
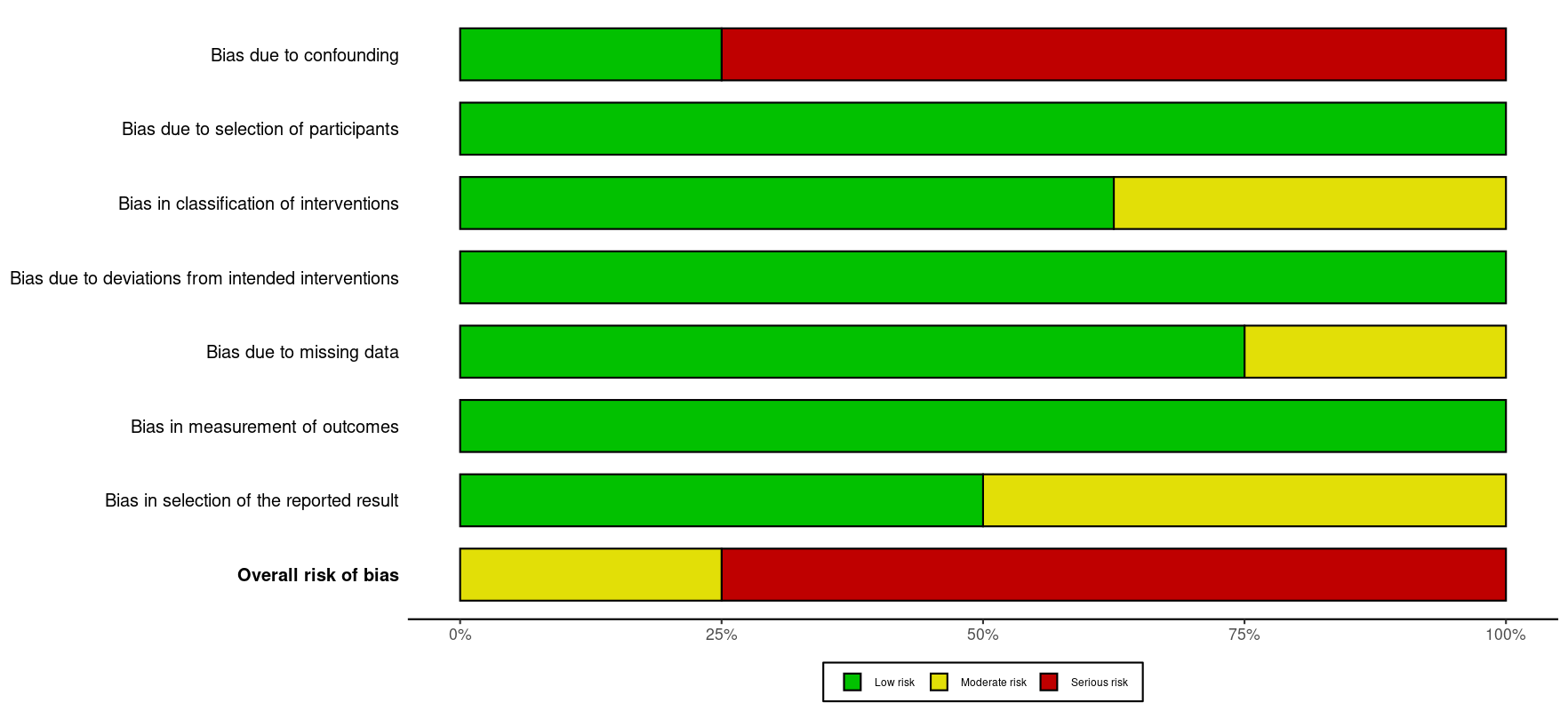
**

**B**

**Figure S2** Overall risk of bias assessed by ROBINS-I tool (A) traffic light plot and (B) summary plot


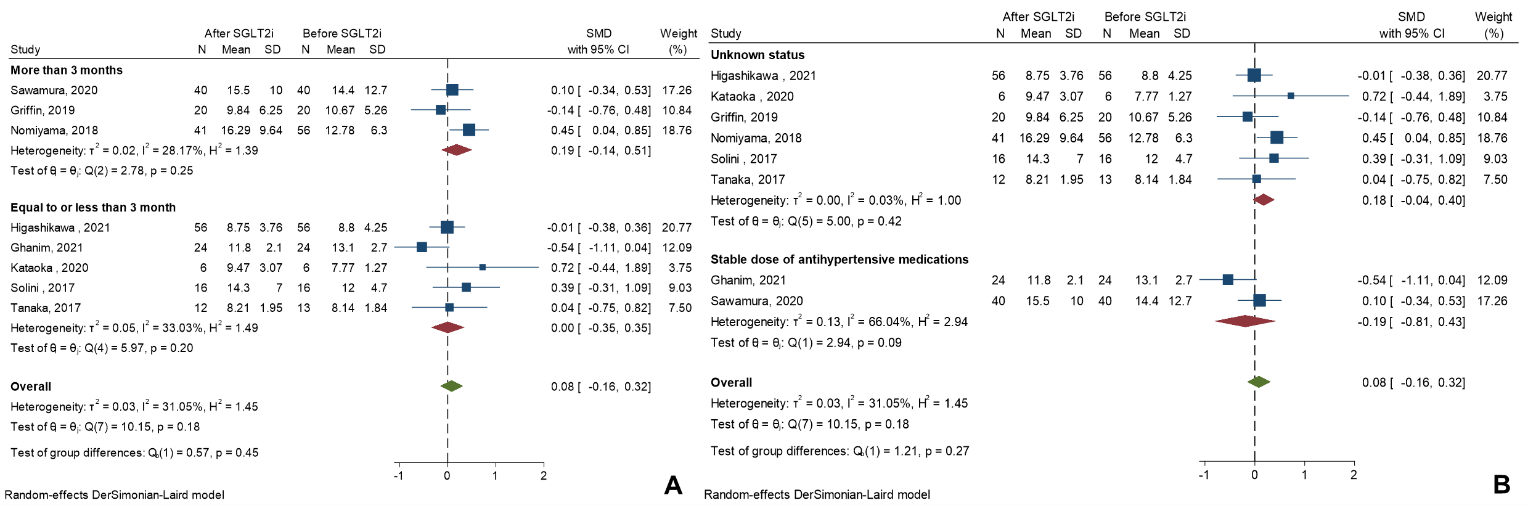


**Figure S3** Forest plots of subgroup analysis of the changes of plasma aldosterone concentration after SGLT2i use in non-randomized controlled studies by (A) duration of SGLT2i use and (B) the stability of the dosage of anti-hypertensive medications during the study


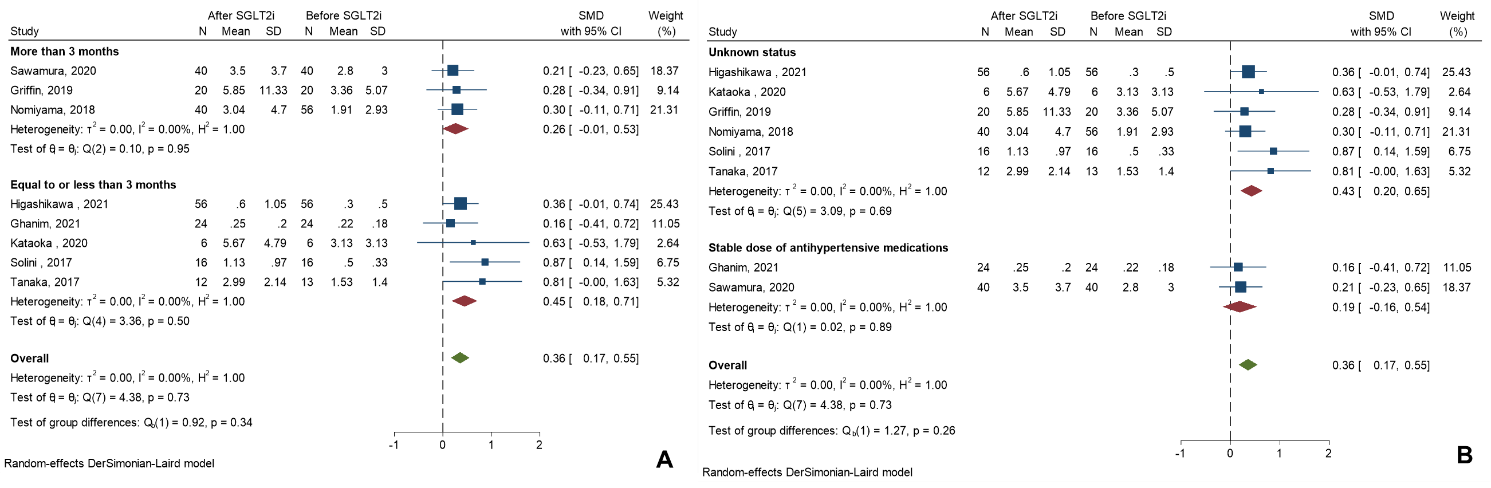


**Figure S4** Forest plots of subgroup analysis of the changes of plasma renin activity after SGLT2i use in non-randomized controlled studies by (A) duration of SGLT2i use and (B) the stability of the dosage of anti-hypertensive medications during the study


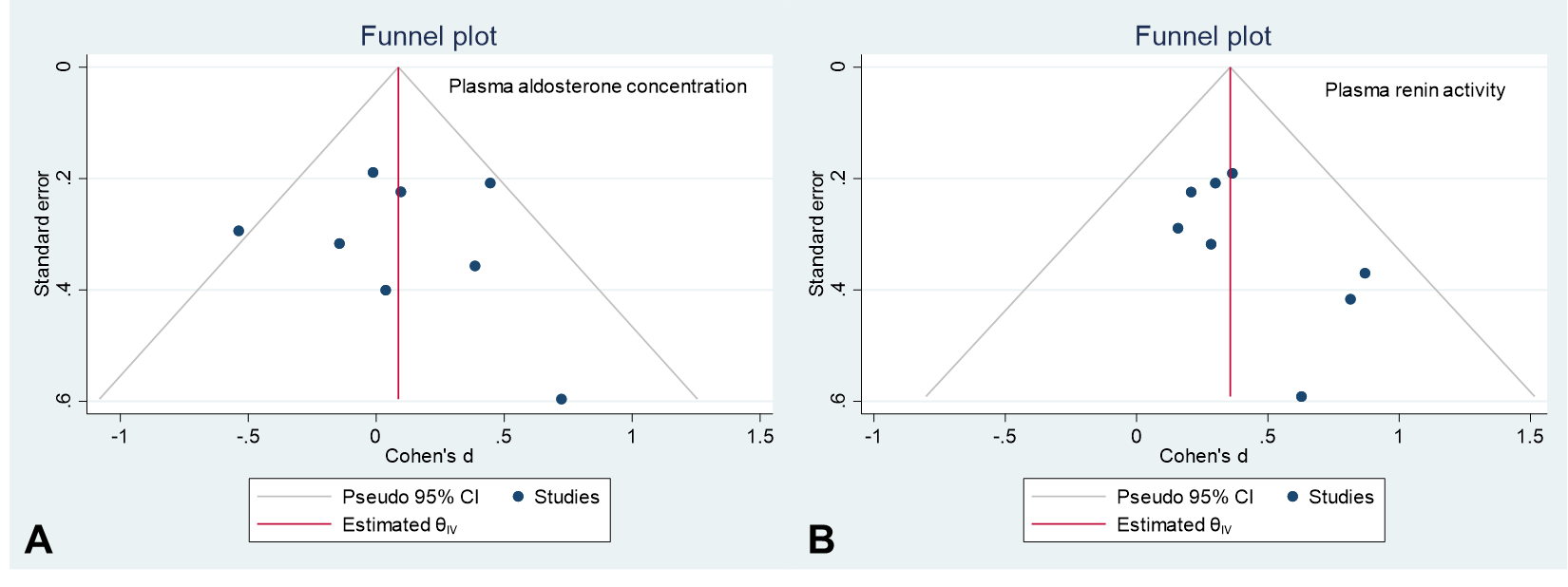


**Figure S5** Funnel plots of non-randomized controlled studies (A) plasma aldosterone concentration (B) plasma renin activity
